# Fe_3_O_4_-PVDF Composite Network for Dendrite-Free Lithium Metal Batteries

**DOI:** 10.3390/nano13202782

**Published:** 2023-10-17

**Authors:** Yun Ou, Chaoyong Ma, Zhiyong Tang, Chenqi Yao, Yunzhuo Zhao, Juanjuan Cheng

**Affiliations:** 1 Hunan Provincial Key Lab of Advanced Materials for New Energy Storage and Conversion, School of Materials Science and Engineering, Hunan University of Science and Technology, Xiangtan 411201, China; 20020601007@mail.hnust.edu.cn (Z.T.); 21011901007@mail.hnust.edu.cn (C.Y.); 22021901004@mail.hnust.edu.cn (Y.Z.); 2 Guizhou Meiling Power Sources Co., Ltd., No. 705 Zhonghua Rd., Huichuan District, Zunyi 563003, China; 20020601010@mail.hnust.edu.cn

**Keywords:** lithium anode, electrospinning, Fe_3_O_4_, PVDF, dendrite-free

## Abstract

Dendrite growth has been the main trouble preventing the practical application of Li metal anodes. Herein, we present how an Fe_3_O_4_-PVDF composite network prepared by using electrospinning has been designed to protect lithium metal anodes effectively. In the symmetrical cells test, the cell with the Fe_3_O_4_-PVDF composite network maintains good cycle performance after 600 h (500 cycles) at a current density of 1 mA cm^−2^ and a plating/stripping capacity of 1 mAh cm^−2^. The bulky Li dendrite is suppressed and a uniform Li deposition remains after long cycling. The characteristics of this engineered separator are further demonstrated in Li-S full cells with a good cycle performance (capacity of 419 mAh g^−1^ after 300 cycles at 0.5 C). This work provides a new idea for the protection of lithium metal anodes.

## 1. Introduction

The lithium metal anode, based on its own ultra-high theoretical specific capacity (3860 mAh g^−1^), low standard electrode potential of −3.04 V, and high energy density, has been developed rapidly [1,2,3]. However, the growth of Li dendrites and the instability of solid electrolyte interphase (SEI) hinder the further practical application of Li metal anodes [4,5,6]. Inhibiting the Li dendrites through the spontaneous reaction of Li metal and electrolytes is of great significance for promoting the practical application of Li anode batteries, with their enhanced safety and extended cycle life [7,8].

To address this challenge, researchers have devised 3D host structures, characterized by porous frameworks and expansive specific surface areas, to serve as matrices for lithium, including graphene oxide [8], nickel foam matrix [9], and nitrogen-doped graphene [10,11]. These porous architectures offer multiple nucleation sites and the capacity to withstand volume fluctuations during lithium plating/stripping. Moreover, their substantial specific surface areas promote a uniform distribution of current density across the electrode surface [12,13]. The combination of numerous nucleation sites and an even current density distribution facilitates long-lasting cycle performance under high current densities. However, 3D structures alone cannot fully mitigate irreversible electrolyte losses and the adjustment of SEI composition during discharge and charge processes [14,15]. Furthermore, these 3D electrodes tend to exhibit increased volume and reduced loading, which limits the continuous enhancement of battery energy density due to long-term irreversible consumption [16]. To overcome these challenges, various strategies have been explored. One approach involves introducing functional additives, such as fluoroethylene carbonate [17,18], lithium nitrate [19], and aluminum chloride, to the electrolyte [20], or directly constructing artificial protection materials, such as lithium fluoride, boron nitride, and lithium nitride, on the surface of lithium metal. Such methods can improve the mechanical properties and ionic conductivity of SEI. However, the artificial protective layer is limited by its specific surface area and cannot achieve stable cycling at high current densities [21]. The intriguing question, then, is how to synergistically combine the strengths of these various approaches to enhance the electrochemical performance of lithium metal anodes [21,22].

It has been reported that the magnetic field can enhance the cycle stability of lithium anodes and prevent the growth of lithium dendrites based on the magnetohydrodynamic (MHD) effect [23,24]. With the presence of a magnetic field, lithium plating becomes more uniform and denser, and the crystals will grow larger. As a result, the use of magnetic fields as a strategy to address the challenge of lithium dendrites in lithium-metal-based rechargeable batteries has been proposed [24,25].

In this study, an Fe_3_O_4_-PVDF composite network prepared by using electrospinning has been designed to protect the lithium metal anode. This composite network offers several advantages: (i) Fe_3_O_4_ establishes a magnetic field inside the battery to promote the uniform deposition of Li metal [26]. (ⅱ) The hydrophobic nature PVDF maintains enhances its compatibility with Li metal and guides the nucleation process of Li metal [27]. (iii) Furthermore, the excellent thermal and high mechanical stability of the composite network also helps to maintain the integrity of the structure.

## 2. Experiment

### 2.1. Fe_3_O_4_-PVDF Synthesis

The preparation of the electrospinning PVDF precursor and Fe_3_O_4_-PVDF precursor was carried out as described in our previous work [28]. First, a PVDF precursor solution was synthesized. A 1.0 g amount of PVDF and 0.8 g of Fe_3_O_4_ were placed in an oven at 80 °C for 1 h. Additionally, 1.0 g of PVDF was added to a mixed solution of 10 mL of DMF and acetone (7:3 *v*/*v*), placed on a magnetic stirrer at room temperature, and stirred for 10 h. Another part of the same PVDF precursor was added to 0.8 g of Fe_3_O_4_ nanoparticles and mechanically stirred (3000 r/min) for 20 min, then removed and left to stand for 5 min. The two polymer solutions were taken up in plastic syringes, respectively, a suitable stainless steel needle was selected according to the required fiber specifications to connect to a high-voltage power supply, and the surface of the receiving plate was covered with aluminum foil as a receiving device for electrospinning. The spinning parameter settings were as follows: voltage 10 KV; constant flow rate 0.3 mL h^−1^; spinning volume 8 mL; 21 G stainless steel needle, 13 cm distance from the receiving plate; temperature and humidity controlled at room temperature and 31–35%, respectively. The samples were dried using a freeze dryer and then were collected and cut into small discs with a diameter of 18 mm for future use. The Fe_3_O_4_-PVDF/PVDF/Fe_3_O_4_-PVDF network was prepared through electrospinning the PVDF solution onto an electrospun Fe_3_O_4_-PVDF network and then electrospinning the Fe_3_O_4_-PVDF solution onto the resulting Fe_3_O_4_-PVDF/PVDF network (Figure S1). Those samples were also then dried using the freeze dryer and cut into small discs with a diameter of 18 mm for further use. For comparison, a film containing a mixture of Fe_3_O_4_-PVDF (CFP) was prepared through a preparation slurry and then scraper coating to achieve the same thickness of the Fe_3_O_4_-PVDF composite network (Figure S1).

### 2.2. Preparation of Sulfur Cathode

To check the effects of the Fe_3_O_4_-PVDF composite network, the sulfur used in the cathode was pure sulfur without a porous carbon matrix. The mixture was weighed (sulfur:acetylene black:sodium alginate = 7:2:1), placed in a mortar and ground for 0.5 h, transferred to a beaker, added to an appropriate amount of 5% isopropanol aqueous solution, and magnetically stirred for 12 h. The slurry was applied onto an aluminum foil using a compact coating machine and subsequently dried under vacuum conditions at 80 °C for a duration of 12 h. The sulfur loading for each electrode was determined to be approximately 1.2 mg cm^−2^.

### 2.3. Structure Characterization

The crystal structure of the prepared samples was characterized using an X-ray diffractometer (XRD, Bruker AXS D8, Bruker AXS GmbH, Karlsruhe, Germany). The surface morphology of samples was analyzed using a scanning electron microscope (SEM MIRA3 TESCAN, TESCAN, Brno, Czech Republic), and the topography as well as 3D structure surface of samples were examined using an atomic force microscope (AFM, Agilent 5500, Agilent, Santa Clara, CA, USA).

### 2.4. Electrochemical Measurement

Assembly procedure for symmetrical cells: In a glove box filled with Ar gas (the water and oxygen concentration were maintained at less than 0.1 ppm), metal lithium was used as the counter electrode of the cell, and PP, PVDF, PVDF/PP/PVDF (PPP), Fe_3_O_4_-PVDF (FP), Fe_3_O_4_-PVDF/PP/Fe_3_O_4_-PVDF (FPPFP-1), and Fe_3_O_4_-PVDF/PVDF/Fe_3_O_4_-PVDF (FPPFP-2) were assembled according to the structure shown in Figure S1. The electrolyte was 1 M bis(trifluoro-methane) sulfonamide lithium (LiTFSI), and 1.0% LiNO_3_ was dissolved in a mixed solvent of DOL and DME at a volumetric ratio of 1:1. The amount of electrolyte added for cycling in each coin cell was constant at 20 μL. Discharge–charge measurement was carried out with a NEWARE system. As shown in Figure S3b, sulfur and lithium metals were used as cathodes and anodes of the cell, and the composite network of PP/Fe_3_O_4_-PVDF (PFP-1) and PVDF/Fe_3_O_4_-PVDF (PFP-2) was used as the separator. Cyclic voltammetry (CV) and electrochemical impedance (EIS) tests were performed using an electrochemical workstation (CHI 660). CV measurements were performed at a voltage range of 1.7–2.8 V (vs. Li^+^/Li) at a scan rate of 0.1 mV s^−1^, and the frequency range of EIS was fixed to be within 10 mHz–100 kHz.

## 3. Results and Discussion

**Figure 1:**
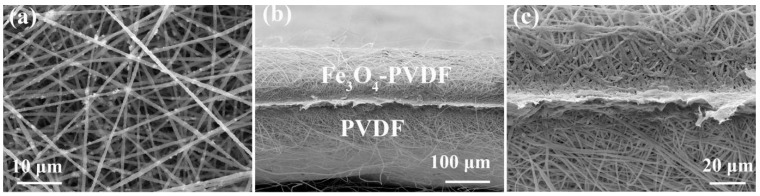
(**a**) A SEM image obtained from the surface of the Fe_3_O_4_-PVDF composite network. (**b**,**c**) The section SEM images of the PVDF/Fe_3_O_4_-PVDF composite network with different magnifications.

The structure of the electrospun Fe_3_O_4_-PVDF composite network is shown in Figure 1. It can be seen that the Fe_3_O_4_ is located within PVDF fibers, and the thickness of the film is about 100 μm. The magnetic field induced with the Fe_3_O_4_-PVDF composite network was reported in our previous work about the PVDF/Fe_3_O_4_ network for a sulfur cathode [28]. The Fe_3_O_4_ nanoparticles are at about 500 nm, which is within the PVDF fibers, and the 3D network interstitial structure with alternating overlapping fibers can effectively cope with the growth of lithium dendrites [29].

**Figure 2:**
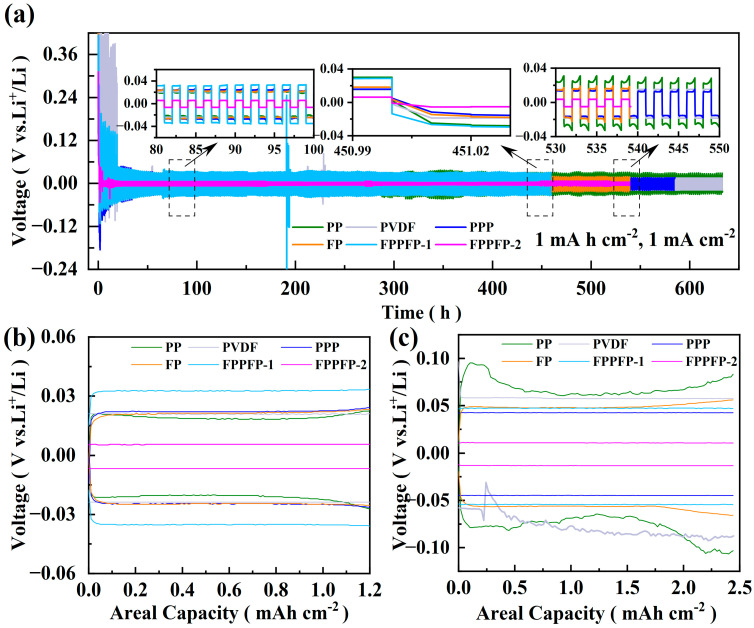
(**a**) Cycle curves of PP, PVDF, PPP, FP, FPPFP-1, and FPPFP-2 at 1 mA cm^−2^ and 1 mAh cm^−2^. (**b**,**c**) The 50th voltage polarization curves of PP, PVDF, PPP, FP, FPPFP-1, and FPPFP-2 symmetrical batteries at 1 mAh cm^−2^, 1 mA cm^−2^.

The possible effects on Li plating/stripping (1 mAh cm^−2^, 1 mA cm^−2^; 2 mAh cm^−2^, 2 mA cm^−2^) from the Fe_3_O_4_-PVDF composite network are demonstrated (Figure 2). The FPPFP-2 (Fe_3_O_4_-PVDF/PVDF/Fe_3_O_4_-PVDF), PVDF, PPP (PVDF/PP/PVDF), FP (Fe_3_O_4_-PVDF), and FPPFP-1 (Fe_3_O_4_-PVDF/PP/Fe_3_O_4_-PVDF) with and without the network can be synchronously compared. With the voltage hysteresis value of the constant current in discharge and charge curves, we can judge the effect of different structures in the long cycle process [30]. As shown in Figure 2, FPPFP-2 exhibits smaller post-voltage and cycle stability compared with others. The cycling performance of symmetrical cells under the conditions of 1 mA cm^−2^ current density and 1 mAh cm^−2^ lithium deposition/desolvation is shown in Figure 2a.

The initial charging overpotentials of the PP and PVDF electrodes were 230 and 365 mV, respectively. Notably, during the subsequent charging process, the overpotential decline exhibited for PVDF electrodes was significantly higher than that of the PP electrodes. A preliminary explanation for this phenomenon is the slower growth rate of lithium dendrites on the surface of PVDF electrodes in comparison to that of PP electrodes [31]. After 460 h of cycling, the overpotentials for the PP, PVDF, PPP, FP, FPPFP-1, and FPPFP-2 electrodes were 28.5, 18.2, 16.1, 19.5, 30.6, and 4.3 mV, respectively. It is evident that, when compared to the other electrodes, the FPPFP-2 electrode demonstrates excellent long-term cycle stability and lower voltage hysteresis. It can be deduced that other electrodes have different degrees of side reactions on the surface of the lithium anode as the cycling progresses. Consequently, the interface impedance between the electrode and electrolyte increases, which leads to different degrees of voltage hysteresis. Remarkably, after 500 h of cycling, the overpotential of the FPPFP-2 electrode was only 3.1 mV. When the current density and deposition/desolvation amount increased to 2 mA cm^−2^ and 2 mAh cm^−2^, the FPPFP-2 electrode was capable of sustaining 300 cycles while maintaining a small overpotential of only 8.3 mV (Figure S2).

Figure 2b,c show the voltage polarization curves for the PP, PVDF, PPP, FP, FPPFP-1, and FPPFP-2 electrodes after 50 cycles at different current densities under different metal deposition/dissolution conditions. With the increment of current density and lithium deposition capacity, the polarization voltages of all six electrodes also increased, but note that the polarization voltages of FPPFP-2 are always lower than others. Figure 2c reveals that at 50 cycles, the electrode lacking the prepared network experiences fluctuating polarization voltage. When mapped to the microcosmic process, this behavior corresponds to the accumulation of a substantial charge in a certain area, resulting in a non-uniform state of current density distribution across the electrode surface, and ultimately triggering the rapid growth of lithium dendrites when lithium deposits in the inner tip protrude [32]. Furthermore, in order to quantify the performance of the Li-Li symmetric cells, critical current density (CCD) tests were conducted under 5/10 mA cm^−2^ and 1 mAh cm^−2^. The results are shown in Figure S4. At a fixed areal capacity of 1 mAh cm^−2^, voltage hystereses of 125 and 275 mV are presented at 5 and 10 mA cm^−2^, respectively, and the cells show a cyclic stability within 30 h.

**Figure 3:**
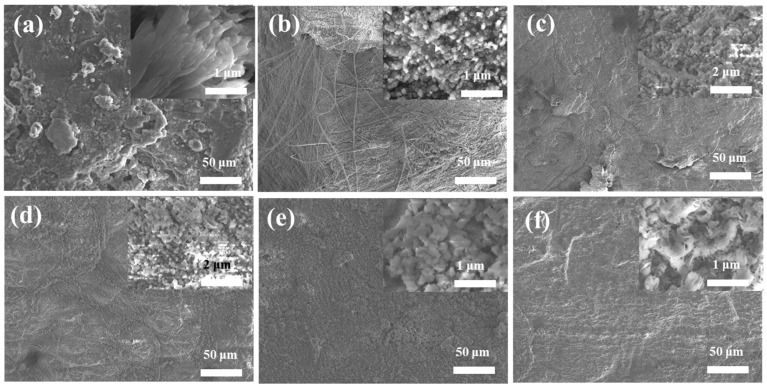
(**a**–**f**) SEM of lithium wafer surface after 250 cycles of symmetric batteries PP, PVDF, PPP, FP, FPPFP-1, and FPPFP-2 at 1 mAh cm^−2^ and 1 mA cm^−2^.

Figure 3 presents the surface morphology of the lithium anode subjected to 250 cycles, both with and without the Fe_3_O_4_-PVDF composite network at a current density of 1 mA cm^−2^ and a capacity of 1 mAh cm^−2^. As shown in Figure 3a, it can be observed that there is vigorous growth of bulky dendrites, which form the terrace structure and the surface structure of the lithium metal electrode without the Fe_3_O_4_-PVDF composite network. Dendrites of the PVDF and PPP (Figure 3b,c) are clustered, forming dense strips with a few regions of reduction on the electrode surface. Upon introducing Fe_3_O_4_, fine lithium particles emerge on the surface of the FP, FPPFP-1, and FPPFP-2 electrodes, creating localized unevenness on the Li surface. It can be seen that the lithium surface displays a significant improvement compared to the PVDF and PPP samples without the composite network. Notably, the FPPFP-1 and FPPFP-2 electrodes consistently maintain a good surface morphology throughout the cycling, exhibiting minimal dendrite formation relative to the FP (Figure 3e,f). The above analysis of the lithium anode’s surface morphology reveals that the electrospun PVDF network effectively inhibits the formation of bulky dendrites. Furthermore, the Fe_3_O_4_-PVDF composite network facilitates uniform lithium deposition while maintaining the integrity of the electrode structure.

**Figure 4:**
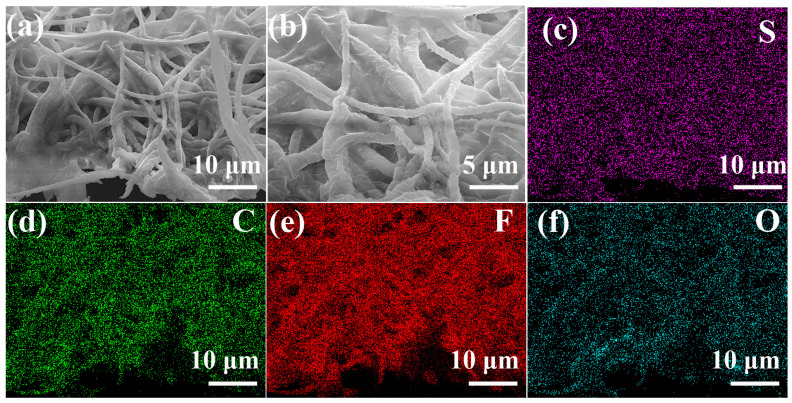
(**a**,**b**) SEM images of the cross-section of FPPFP-2 post-lithium plating and (**c**–**f**) the corresponding SEM-EDX mapping.

To clarify whether lithium is deposited at unintended locations on or within the Fe_3_O_4_-PVDF composite network film, Li deposition in Li-Li symmetric cells was conducted under the conditions of 1 mA cm^−2^ current density and 1 mAh cm^−2^ lithium deposition/desolvation. After cycling, SEM images of the cross-section of FPPFP-2 post-lithium plating and the corresponding SEM-EDX oxygen signal mapping were tested (Figure 4). From Figure 4a,b, it can be seen that the post-lithium plating network is integrated and smooth, which will provide a feasible environment for Li deposition. Figure 4c–f show the SEM-EDX results of carbon, fluorine, sulfur, and oxygen. The carbon and fluorine elements come from the PVDF, and sulfur comes from the electrolyte. Oxygen mapping is similar to that of carbon and fluorine. Oxygen is about 6.4 wt.% (Figure S5), indicating limited lithium located on the Fe_3_O_4_-PVDF composite network film.

Atomic force microscopy (AFM) was used to observe the 3D morphology of lithium metal and calculate the depth of the region. Figure 4 shows the AFM images of Li anodes with and without the Fe_3_O_4_-PVDF composite network after 250 cycles at a current density of 1 mA cm^−2^ and a capacity of 1 mAh cm^−2^.

**Figure 5:**
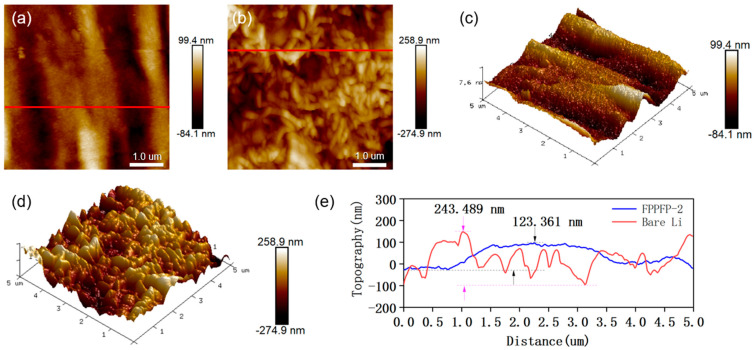
AFM test for the PP and FPPFP-2 electrodes after 250 cycles at a current density of 1 mA cm^−2^ and a capacity of 1 mAh cm^−2^. (**a**,**b**) Planar AFM images for the PP and FPPFP-2 electrodes; (**c**,**d**) 3D AFM images of the PP and FPPFP-2 electrodes; (**e**) height difference of the PP and FPPFP-2 electrodes.

In Figure 5a, it can be seen that the coarse metal particles and moss-like dendrites on the surface of the Li anode without the Fe_3_O_4_-PVDF composite network are widely distributed on the surface of the lithium metal, forming a hilly and mountainous topography, akin to a concave–convex structure (3D diagram). This indicates that the metal surface has undergone significant degradation due to the growing lithium dendrites. Continued use of the electrode aggravates metal corrosion and electrolyte consumption, and the dendrites continue to grow in the form of whiskers and dendrites, potentially initiating unforeseen side reactions [33]. In contrast, the FPPFP-2 electrode presents a uniform grain size and a flat distribution, with slight fluctuations in certain regions from a two-dimensional plan view. In the 3D overall view, the electrode surface is smooth and flat, which proves that Fe_3_O_4_-PVDF has a positive effect on inhibition of lithium dendrites. To further show the inhibitory effect of the Fe_3_O_4_-PVDF composite network on lithium dendrites, a height difference curve was drawn from a randomly selected area, which indicates that the surface relief of FPPFP-2 is smaller, measuring only 123.4 nm (Figure 5e).

To demonstrate the effect of the network in the Fe_3_O_4_-PVDF composite, the CFP were assembled (as shown in Figure S1) to form Li||CFP||Li symmetrical cells and cycled under 1 mA cm^−2^ 1 mAh cm^−2^ and 2 mA cm^−2^ 2 mAh cm^−2^, respectively. The surface morphology of the cycled Li was examined via SEM, as presented in Figure S3. The investigation revealed that the lithium dendrites appear to exist in a needle-like or linear, one-dimensional, fibrous structure at low current density, and the lithium ion transport is limited by the current density (Figure S3a). As the current density increased, lithium ions were further consumed. At this time, the concentration gradient field on the dendrite surface becomes uncertain due to the influence of the limited consumption of lithium ions. This uncertainty prompts dendrites to extend randomly, resulting in the formation of a productive, mossy structure [34]. Throughout the discharge and charge progress, the moss-like surface provides active sites for direct reaction between lithium metal and electrolyte, thereby causing the cluster-like moss structure to evolve into a bush-like dendritic structure (as shown in Figure S3b).

In Figure S6a, a comparison is made between the Li^+^ deposition process with and without magnetic field. Due to the tiny protrusions on the surface of the lithium metal, when the electric force lines approach these areas with high curvature radius, they assume a non-uniform distribution state, resulting in the disruption of the electric field distribution [12]. When Li^+^ is deposited, Li^+^ tends to accumulate at the tip of the surface, resulting in an extremely inhomogeneous distribution of ion concentration on the lithium surface, and the continuous deposition exacerbates the rapid growth of lithium dendrites [32]. When a parallel magnetic field is introduced, Li^+^ diffusion toward the protrusions causes the moving Li^+^ to cut the magnetic field line to generate Lorentz force, and this force perpendicular to the magnetic field and electric field continuously alters the moving direction of Li^+^ and induces convection within the electrolyte. Consequently, Li^+^ ions are uniformly distributed on the Li metal surface [35]. Finally, the problem of lithium dendrite growth is solved, enabling Li^+^ to form a dense and uniform deposit layer on the surface.

To verify the application potential of the Fe_3_O_4_-PVDF composite network, we assembled PFP-1 and PFP-2 into the Li-S cells according to the diagram depicted in Figure S6b and conducted galvanostatic charge and discharge testing.

The battery capacity is preliminarily determined by the size of the closed curve area surrounded by the cyclic voltammetry (CV) curve. It can be seen from Figure S7 that both the cyclic voltammetry curves of PFP-1 and PFP-2 exhibit the typical redox peaks characteristic of Li-S batteries. Notably, these CV curves have a strong reduction peak at 2.35 V and 2.04 V (vs. Li^+^/Li), respectively, along with a strong oxidation peak around 2.45 V. The reduction peak at 2.35 V corresponds to the reaction process in which the S_8_ ring undergoes disintegration, forming long-chain polysulfide ions. Simultaneously, the reduction peak at 2.04 V represents the reaction process where long-chain polysulfides further oxidize to form short chains and ultimately yield Li_2_S. The reduction peak near 2.45 V corresponds to two reverse reduction processes at 2.35 V and 2.04 V [36,37]. It can also be found from Figure S7b that, compared with the 5-cycle CV curves of PFP-1, those of PFP-2 are almost overlapped, indicating that the PFP-2 separator has good electrochemical reversibility after being applied to Li-S batteries [38]. It is obvious that the battery polarization phenomenon is weakened in the second and subsequent cycles, and the reduction peak moves to the high-voltage side. These results show that the PVDF framework is beneficial to weakening the polarization of the sulfur cathode as well as improving the cycling stability of the electrode [39]. This conclusion is further supported by the comparison of electrode cycle performance.

**Figure 6:**
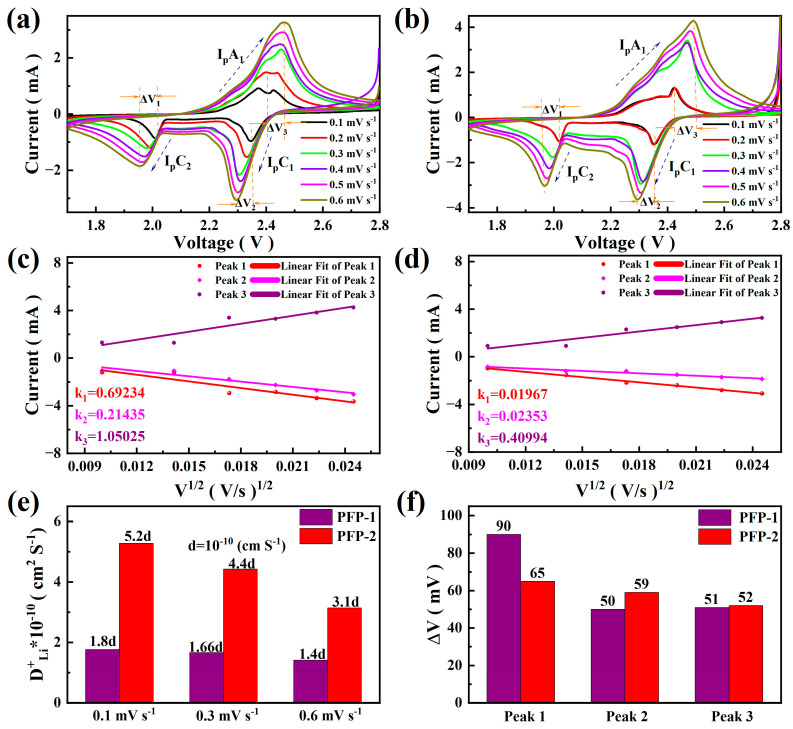
(**a**,**b**) Cyclic voltammograms of PFP-1 and PFP-2 at different scan rates; (**c**,**d**) linear comparison of current and scan rate between PFP-1 and PFP-2; (**e**,**f**) comparison of ion diffusion coefficient with redox peak for PFP-1 and PFP-2.

Enhancing the contact surface area between the separator and cathode material can effectively improve the Li^+^ transport efficiency of the separator during electrochemical reaction. As shown in Figure 6, cyclic voltammetry tests were performed on PFP-1 and PFP-2 at a scan rate ranging from 0.1 to 0.6 mV s^−1^. Through these tests, it was found that as the scan rate increased, with the influence of the ion transmission rate, the redox peaks associated with the two diagrams corresponding to the cell showed different degrees of positive and negative shifts, which made the test cell produce a certain degree of polarization at higher scan rates.

Compared with PFP-1, PFP-2 exhibits smaller polarization voltage. The CV tests performed at different sweep speeds confirm that the presence of PVDF frameworks enhances the Li^+^ transmission rate at the interface between the positive electrode and the separator. Consequently, the lithium insertion and delithiation of the battery can be improved [40], while the kinetics of the process are significantly enhanced (Figure 6e).

Utilizing the relationship between the positive and negative peak current intensity and the square root of the scan rate, we applied the same Randles–Sevcik equation as Formula (1) to quantitatively evaluate the electrochemical performance of PVDF frameworks on the cathode/separator interface. The calculation results are shown in Figure 6f. Compared with PP, PVDF exhibits a substantially enhanced lithium ion diffusion coefficient, which can be explained in that the PVDF framework has greatly improved the ion transport channel and the infiltration of the electrolyte [41].

(1) $$I_{p}=2.69\times 10^{5}n^{1/2}AD_{\mathrm{Li}}^{1/2}V^{1/2}C_{\mathrm{Li}}$$

In the formula, *I*_p_ represents the peak current of CV, *n* signifies the number of electrons engaged in the reaction, *A* represents the electrode area, *D*_Li_ stands for the ion diffusion coefficient, *V* denotes the scan rate, and *C*_Li_ represents the lithium ion concentration within the electrolyte.

**Figure 7:**
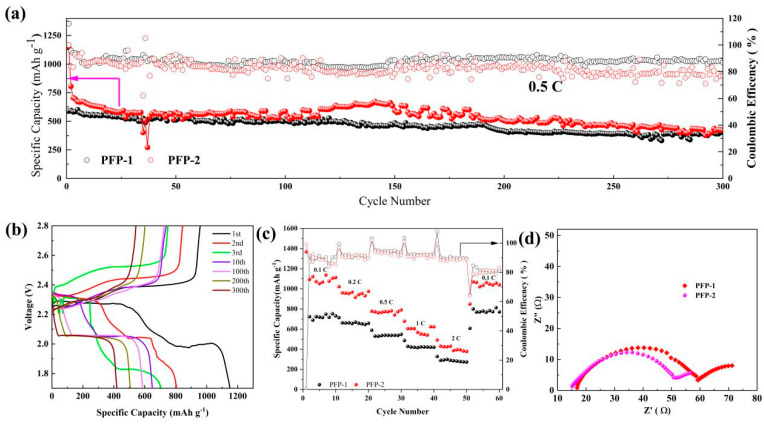
Electrochemical performance of PFP-1 and PFP-2: (**a**) cycling performance at 0.5 C current density; (**b**) charge and discharge profiles of 1st, 2nd, 3rd, 10th, 100th, 200th, and 300th cycles; (**c**) rate performance at different current densities; (**d**) impedance measurement.

Figure 7a shows the cycling performance of Li-S batteries with PFP-1 and PFP-2 at a current density of 0.5 C for 300 cycles. The battery employing PFP-2 has higher initial capacity and better cycle stability in comparison to the PFP-1 battery. After 300 cycles, the retained specific capacities of PFP-1 and PFP-2 are 391 and 419 mAh g^−1^, respectively. This excellent cycle performance of PFP-2 can be attributed to the unique advantages stemming from the excellent mechanics and flexibility of the Fe_3_O_4_-PVDF composite network [42]. For the cathode in the study, sulfur is used without any constrained matrix. In the initial six cycles, the Coulombic efficiency is higher than 92%. The initial higher Coulombic efficiency is related to the large ratio between sulfur loading and the amount of electrolyte. From the 7th cycle to the 300th, the Coulombic efficiency decreased from about 90% to 80%. In Figure 7b, the “shuttle effect” can also be seen. During the long cycle, there are no constrained matrices for lithium polysulfides within the cathode; thus, the dissolution of lithium polysulfides is unavoidable, despite the use of lithium nitrate within the electrolyte [43,44].

The advantages of PFP-2 batteries over PFP-1 are further confirmed when subjected to varying current densities (Figure 7b). After 10 cycles, PFP-2 retains remarkable specific capacities of 1111.7 mAh g^−1^ (at 0.1 C) and 976.4 mAh g^−1^ (at 0.2 C). Upon returning to 0.1 C after 50 cycles, PFP-2 exhibits a reversible capacity of 1039.8 mAh g^−1^, which is 1.25 times that of PFP-1 (801.1 mAh g^−1^), indicating excellent cycle reversibility. The sustained performance and resilience of PFP-2 at high current densities make it a suitable material for lithium anode protection under high power conditions. Moreover, PFP-2 holds an additional advantage over PFP-1 in terms of a better liquid absorption rate, promoting more comprehensive electrolyte contact with the electrode and reducing charge transfer resistance (R_ct_) (Figure 7c). It can be concluded that the Fe_3_O_4_-PVDF composite network has a better effect when combined with the PVDF separator.

## 4. Conclusions

We successfully developed an electrospun Fe_3_O_4_-PVDF composite network, which effectively inhibits the growth of lithium dendrites. Involving Fe_3_O_4_ establishes a built-in magnetic field that ensures the uniform distribution of Li^+^ and the good mechanical properties of the PVDF network, which ensures the integrity of the electrode. In comparison to PP, the Fe_3_O_4_-PVDF composite network integrated into the PVDF separator demonstrates a longer cycle life and lower lithium ion deposition potential. In contrast, the coated Fe_3_O_4_ and PVDF do not provide as effective protection for the lithium anode. Therefore, it is evident that the Fe_3_O_4_-PVDF composite network exhibits superior performance when engineered on PVDF rather than on PP.

## Data Availability

The data presented in this study are available on request from the corresponding author.

## Supplementary Materials

The following supporting information can be downloaded at: https://www.mdpi.com/article/10.3390/nano13202782/s1, Figure S1: Schematic diagram of battery assembly structure and corresponding abbreviations; Figure S2: Cycling performance curves of PP, PVDF, PPP, FP, FPPFP-1, FPPFP-2 symmetrical cells at 1 mA cm^−2^ 1 mA h cm^−2^; Figure S3: Li dendrite deposition of CFP after cycling at (a) 1 mA cm^−2^ 1 mA h cm^−2^ and (b) 2 mA cm^−2^ 2 mA h cm^−2^; Figure S4: (CCD) tests conducted under 5/10 mA cm^−2^ and 1 mAh cm^−2^; Figure S5: Automatic peak identification of carbon, fluorine, sulfur, and oxygen in EDS spectrum; Figure S6: (a) Schematic diagram of lithium dendrite deposition, (b) Schematic diagram of the structure of the full battery; Figure S7: Cyclic voltammetry curves of PFP-1 and PFP-2 full batteries.
