# Supplementary material for: Fe3O4-PVDF Composite Network for Dendrite-Free Lithium Metal Batteries
Source: Nanomaterials (Basel). 2023 Oct 17;13(20):2782. doi: 10.3390/nano13202782 (PMC10609358; doi:10.3390/nano13202782)
Supplement: Supplementary file 1 [file nanomaterials-13-02782-s001.zip › nanomaterials-2578351-supplementary.pdf]

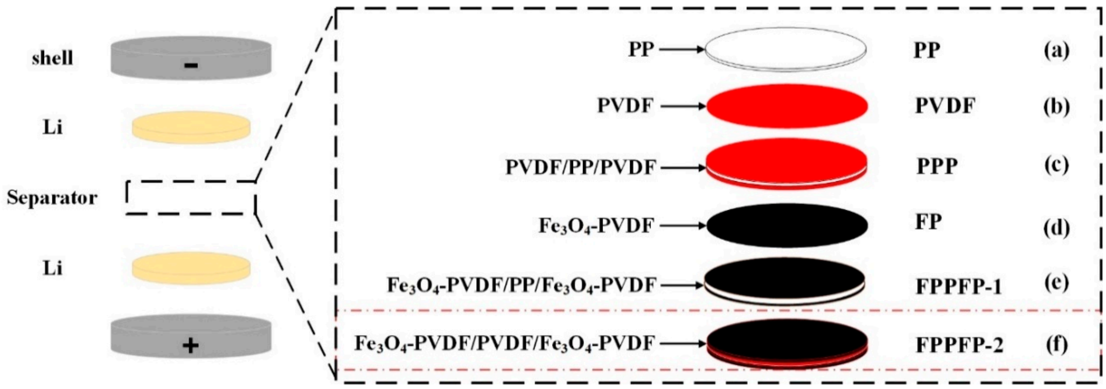

Figure S1 Schematic diagram of battery assembly structure and corresponding abbreviations

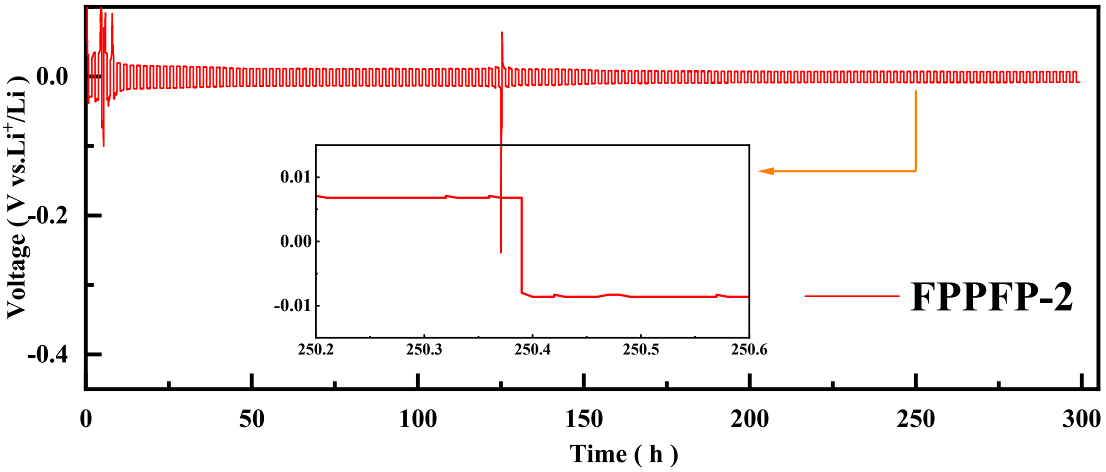

Figure S2 Cycling performance curves of PP, PVDF, PPP, FP, FPPFP-1, FPPFP-2 symmetrical cells at 1 mA cm^{-2} 1 mA h cm^{-2}.

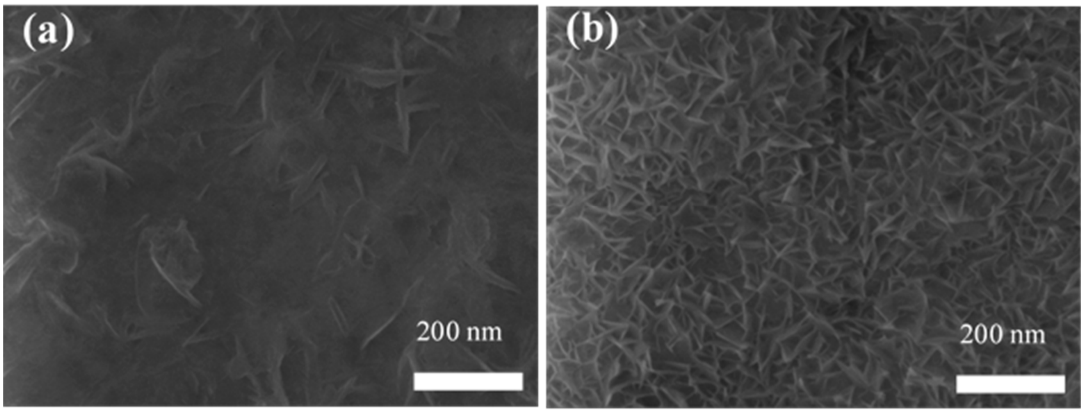

Figure S3 Li dendrite deposition of CFP after cycling at (a)1 mA cm^{-2} 1 mA h cm^{-2} and (b) 2 mA cm^{-2} 2 mA h cm^{-2}

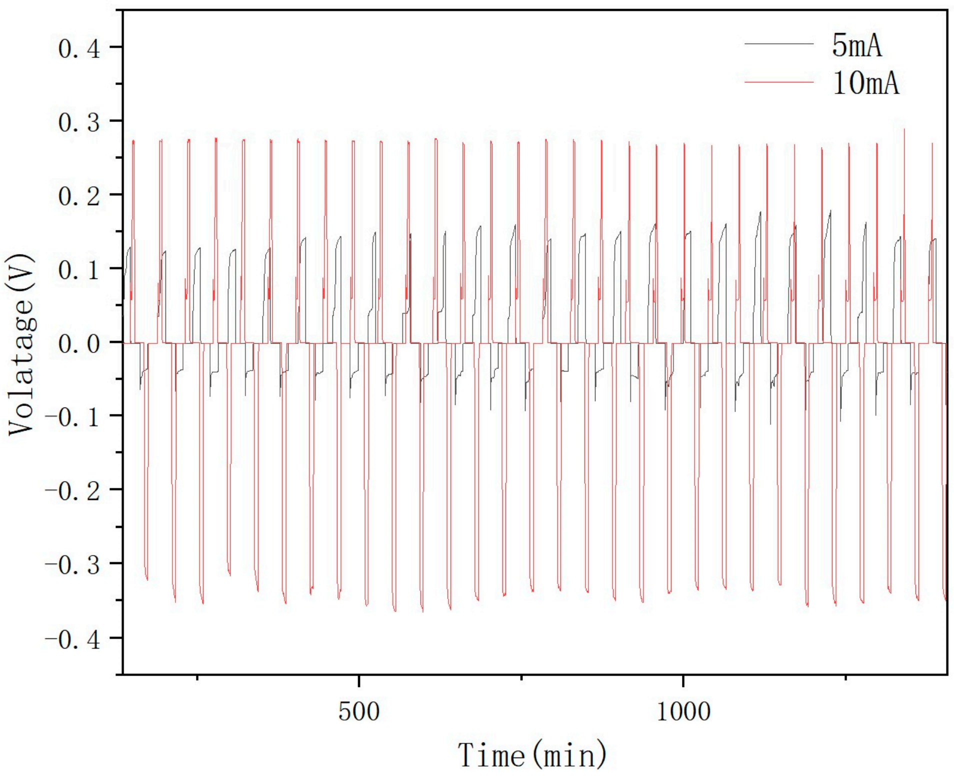

Figure S4 (CCD) tests conducted under 5/10 mA cm^{-2} and 1 mAh cm^{-2}

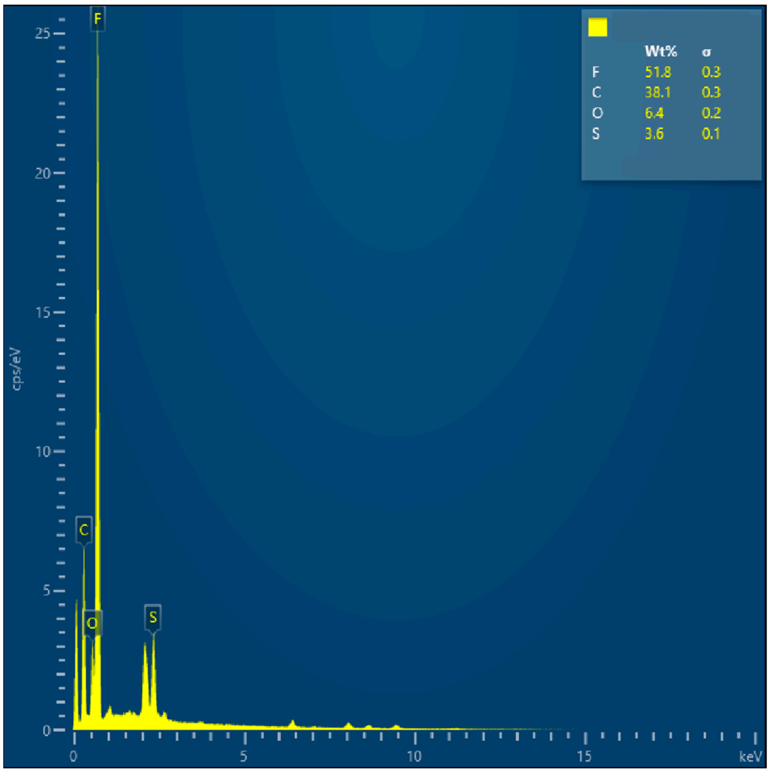

Figure S5 Automatic peak identification of carbon, fluorine, sulfur, and oxygen in EDS spectrum

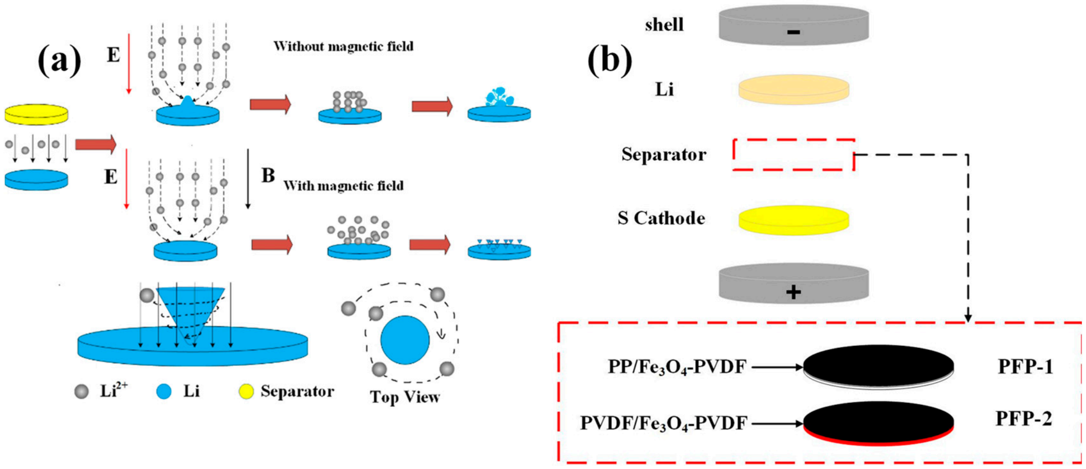

Figure S6 (a)Schematic diagram of lithium dendrite deposition, (b) Schematic diagram of the structure of the full battery

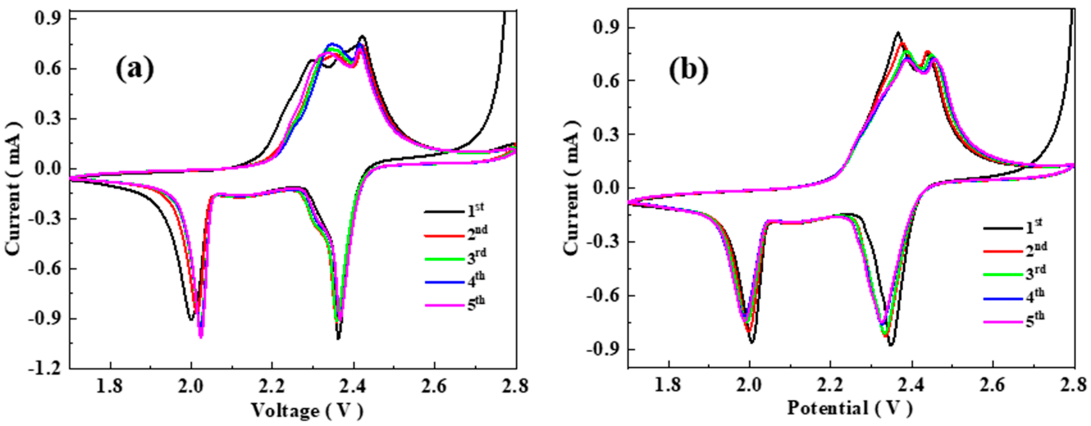

Figure S7 Cyclic voltammetry curves of PFP-1 and PFP-2 full batteries
